# Respectful maternity care and associated factors among women who delivered at Harar hospitals, eastern Ethiopia: a cross-sectional study

**DOI:** 10.1186/s12884-020-2757-x

**Published:** 2020-02-10

**Authors:** Agegnehu Bante, Kedir Teji, Berhanu Seyoum, Abera Mersha

**Affiliations:** 1 Department of Nursing, College of Medicine and Health Sciences, Arba Minch University, Arba Minch, Ethiopia; 2 School of Nursing and Midwifery, College of Health and Medical Sciences, Haramaya University, Harar, Ethiopia; 3 Department of Medical Laboratory Sciences, College of Health and Medical Sciences, Haramaya University, Harar, Ethiopia

**Keywords:** Respectful maternity care, Disrespect, Abuse, Harar, Ethiopia

## Abstract

**Background:**

In Ethiopia, approximately three-fourths of mothers do not deliver in health facilities. Disrespect and abuse during childbirth fallouts in underutilization of institutional delivery that upshots maternal morbidity and mortality. Thus, the ambition of this study was to assess respectful maternity care and associated factors in Harar hospitals, Eastern Ethiopia.

**Methods:**

A facility-based cross-sectional study was conducted from April 01 to July 01, 2017. A total of 425 women, delivered at Harar town hospitals, were nominated using a systematic random sampling technique. A pretested and organized questionnaire was used to collect the data. After checking for completeness, the data were entered into EpiData version 3.1 and exported to SPSS version 22.0 for cleaning and analyses. Both bivariate and multivariable logistic regression was computed to identify factors associated with respectful maternity care. Statistical significance was declared at a *P*-value of < 0.05.

**Results:**

Data were collected on 425 women. Overall, only 38.4% (95% CI: 33.7, 42.0%) of women received respectful maternity care. Delivering at private hospitals [AOR: 2.3, 95% CI: 1.25, 4.07], having ANC follow-up [AOR: 1.8, 95% CI: 1.10, 3.20], planned pregnancy [AOR: 3.0, 95% CI: 1.24, 7.34], labor attended by male provider [AOR: 1.8, 95% CI: 1.14, 2.77] and normal maternal outcome [AOR: 2.3, 95% CI: 1.13, 4.83] were significantly associated with respectful maternity care.

**Conclusions:**

Only four out of ten women received respectful care during labor and delivery. Providing women-friendly, abusive free, timely and discriminative free care are the bases to improve the uptake of institutional delivery. Execution of respectful care advancement must be the business of all healthcare providers. Furthermore, to come up with a substantial reduction in maternal mortality, great emphasis should be given to make the service woman-centered.

## Background

Although there is a momentous reduction, maternal mortality ratio (MMR) is still 239 and 12 per 100,000 live births in developing and developed countries respectively [1]. Regardless of an enlightened improvement in maternal and child health services in Ethiopia; nearly three-fourths of mothers do not deliver in health facilities and do not attend by skilled birth attendants (SBA). Moreover, the MMR is still 412/100,000 live births [2]. This figure is very far from the target under the Sustainable Development Goal (SDG) by 2030, which is < 70/100,000 [3]. Absence of companion during labor, substandard maternity service, long waiting time to receive care, and disrespect and abuse during childbirth are some of the factors that contribute to the underutilization of facility delivery [4–7].

Respectful maternity care (RMC) during childbirth is an interaction between the client and the healthcare providers (HCPs) or facility conditions. It has a significant role in MMR reduction by enhancing clients’ inclination to deliver in health facilities [8–13]. Furthermore, RMC is the standard of care for all women [14] that encompasses women’s basic human rights [9, 15].

In spite of every woman’s right to get the highest achievable standard of care, many women experience disrespect and abusive treatment. Disrespect and abuse (D&A) are often multi-factorial and perceived differently and sometimes it may be considered as normal depending on the context [15–18]. Browser and Hill identified seven categories of disrespect which include: physical abuse, non-consented clinical care, non-confidential care, non-dignified care, discrimination based on specific patient attributes, abandonment of care, and detention in facilities [9].

Maternal health experts and concerned stakeholders agreed that D&A in facility-based childbirth are causes of suffering for women, the barrier to skilled care utilization and often a violation of women‘s human rights  [9]. In addition, a growing body of the literature showed that any childbearing woman that faces D&A results in poor maternal and neonatal outcome [19].

D&A breaks the trust between women and HCPs and may deter women from accessing healthcare services [20]. Abusive free care is not well realized in many parts of the world [21]. Evidence from India, Tanzania, Kenya, and Ethiopia showed that the status of RMC ranges from 21 to 80%. Delivering at night time, private health facilities, cesarean delivery, continuous emotional support during labor, intention to use the facility, assurance of privacy and high economic status were some of the factors associated with RMC [7, 22–25].

Promoting institutional delivery without the implementation of RMC may paradoxically increase maternal mortality [26]. Reevaluating women’s childbirth experience is indispensable to fascinate women with facility delivery [27, 28]. Despite many published evidence about the individual cases of D&A, there is a scantiness of proof concerning the prevalence estimate of RMC in facility-based childbirth; particularly there is a scarceness of evidence in Eastern Ethiopia. Hence, this study was aimed to assess the status of RMC and associated factors at Harar town hospitals, Eastern Ethiopia.

## Methods

### Study setting, design and period

A facility-based cross-sectional study was conducted from April 01 to July 01, 2017, at Harar town hospitals, Eastern Ethiopia. Harar is found 526 km away from Addis Ababa, the capital city of Ethiopia. In view of the 2007 Census projection, the all out populace of the Harari region was 232,000, of which female records 115,230 [29]. Harar town had 19 kebeles (most minimal regulatory unit) separated into six districts. In the town, there were seven hospitals (2 open, 2 private, 1 Nongovernmental (NGO) and 2 different hospitals), 8 health centers and 26 health posts. The antenatal care (ANC), health facility delivery and SBA coverage of the Region reach 75.9, 50.2, and 51.2% respectively [2].

### Study population

Women who visited the nominated Harar hospitals for labor and delivery throughout the information assortment period were included. Women who were fundamentally sick, and unfit to impart were excluded from the study.

### Sample size determination and sampling procedure

A single population proportion formula was used, to determine the minimum adequate sample for this study, with the following assumptions: *P* = 0.214 (proportion of women who received respectful and abusive free care in Addis Ababa) [7], 95% degree of certainty, 4% margin of error and 5% contingency. In view of the above suppositions, the final sample size was 425. Four out of seven hospitals were chosen (i.e. Hiwot Fana Specialized University Hospital (HFSUH), Jugal, Yemage and Harar General hospital) purposively dependent on administration arrangement to people in general and arrangement of essential obstetrics and infant care. At that point, the sample was apportioned relatively to every hospital by investigating the number of deliveries in the earlier year. The information was gathered from every other postnatal woman until the anticipated sample was accomplished.

### Data collection tool

A pretested and organized questionnaire was sorted out from previous literature. The instrument was first arranged in English and converted into nearby dialects (i.e. Amharic and Afaan Oromo) and back to English by free language specialists. The tool incorporates socio-demographic and obstetric characteristics, companion during labor, length of hospital stay, and RMC assessment items. The Maternal and Child Health Integrated Program (MCHIP) developed 23 items in seven categories of D&A as part of the RMC tool kit [30]. However, this tool is not validated in the context of developing countries including Ethiopia. Hence, the level of RMC was assessed using 15 validated items in the Ethiopian context. The tool had four categories with verification items (i.e. friendly care by 7 items, abusive free care using 3 items, timely care using 2 and discrimination-free care using 3 items). The items have a good internal correlation (Cronbach’s alpha (α) of 0.857) and adequate reliability (α = 0.845) [31]. In addition, the tool was pretested on 5% of the sample and alteration was done preceding the beginning of the genuine information assortment.

### Data collectors and data collection procedure

Four qualified diploma birthing assistance experts who were conversant in local dialects and didn’t work in the investigation settings were enlisted for information assortment. The information assortment was directed in an institution based discharge interview using an organized questionnaire regulated poll. Women who satisfy the consideration criteria and ready to partake were met in a private setting after discharge from the postnatal ward. Supervision was carried out on a daily basis throughout the study period.

### Study variables and measurements

Respectful maternity care refers to care organized for and provided to all women in a manner that maintains their dignity, privacy, and confidentiality, ensures freedom from harm and mistreatment and enables informed choice and continuous support during labor and childbirth [32]. RMC, which is a composite variable of four constructs: friendly, abusive free, timely and discrimination-free care was the dependent variable. The level of RMC was assessed using a five-point Likert scale ([1] strongly disagree to [5] strongly agree) questionnaires. The overall cutoff-point for RMC was determined based on previous studies and those participants who encountered D&A for at least one of the 15 items were categorized as “disrespected” [7]. The socio-demographic and obstetric characteristics, companion during labor, and length of hospital stay were the independent variables for this study.

### Data quality assurance

The instrument was pretested on 20 postnatal women at HFSUH one month preceding the actual information assortment. A 2-days preparation covering both hypothetical and commonsense parts of information assortment was held. Close supervision was done consistently all through the investigation time frame. Double data entry was done on 5% of the sample to guarantee the consistency of the entered information.

### Data processing and analysis

After checking completeness, the data were entered into EpiData version 3.1 statistical software. Then, the data were exported to SPSS version 22.0 for cleaning and analysis. During analysis, the reactions of ‘strongly agree’ and ‘agree’ were classified as “Yes” (received RMC) and reactions of ‘strongly disagree’, ‘disagree’ and ‘neutral’ as “No” (disrespected and abused) for positive statements. Those contrarily articulated statements were categorized in the turnaround heading as “strongly disagree” and “disagree” categorized as “Yes” and the reaction of “neutral”, “agree” and “strongly agree” categorized as “No”. Descriptive summary measures such as frequency, percentages, mean and standard deviation were used to describe characteristics of the participants. Binary logistic regression was carried out to identify the factors associated with RMC. To control possible confounding factors, variables with a *p*-value of ≤0.25 in the bivariate analysis were taken to the multivariable analysis. Multicollinearity and model fitness was checked using standard error and Hosmer-Lemeshow test respectively. The adjusted odds ratio (AOR), with 95% confidence intervals (CI), was used to identify the independent variables associated with RMC. Statistical significance was declared at a *P*-value of < 0.05.

## Results

### Socio-demographic characteristics

In this study, 425 women were involved, with a response rate of 100%. The mean (±SD) age of the participants was 27 (±6.0) years. Among the participants, 48% were within the age group of 25–34 years, 63.0% were Oromo by ethnicity, 80.9% were muslims, 98.6% were married and 79.3% were housewives. Of the participants, 39.5% were uneducated and 51.3% were from urban settings (Table 1).

**Table 1:** Socio-demographic characteristics of participants at Harar hospitals, Eastern Ethiopia, 2017 (*N* = 425)

Variables		Frequency	Percentage (%)
Age	< 25	152	35.8
	25–34	204	48.0
	≥35	69	16.2
Ethnicity	Oromo	268	63.0
	Harari	112	26.4
	Amhara	29	6.8
	Other^a^	16	3.8
Religion	Muslim	344	80.9
	Orthodox	76	17.9
	Protestant	5	1.2
Marital status	Married	419	98.6
	Other^b^	6	1.4
Level of education	Unable to read and write	168	39.5
	Able to read and write	50	11.8
	Primary	99	23.3
	Secondary	63	14.8
	College and above	45	10.6
Occupation	Housewife	337	79.3
	Merchant	39	9.2
	Government employee	26	6.1
	Non-government employee	15	3.5
	Other^c^	8	1.9
Residence	Urban	218	51.3
	Rural	207	48.7

^a^Tigray, Gurage, Somali; ^b^widowed, separated, single; ^c^student, daily laborer

### Obstetrics characteristics

**Fig. 1:**
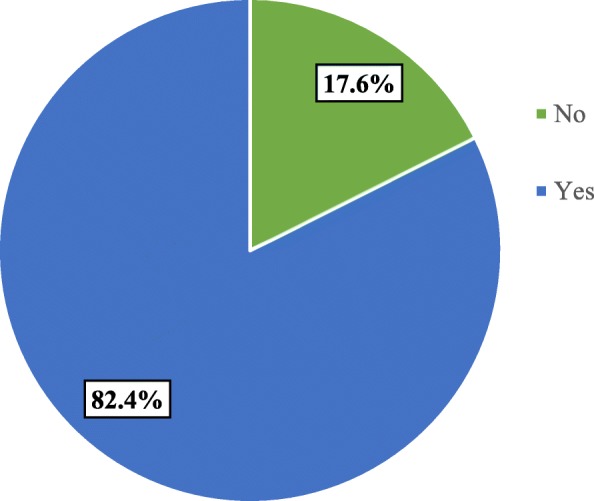
Women’s willingness to visit again and recommend Harar hospitals, Eastern Ethiopia, 2017 (*N* = 425)

More than two-thirds (68%) of women were multipara. Three hundred three (71%) of women had ANC follow-up for the current pregnancy; of them, 58% had four ANC visits and above. Of the participants, 38% came to the hospital through referral and pregnancy was planned for 89% of women. Labor was not attended by a companion for 79% of women and half (49.9%) of the participants were attended by female HCPs. Episiotomy was done for 32% of the participants. Among the participants, 53% were delivered through spontaneous vaginal delivery followed by instrument-assisted delivery (25%). Childbirth ends up without an impediment for 87% of the clients (Table 2). Of the participants, 82.4% were enthusiastic to recommend and visit the facility in the future (Fig. 1).

**Table 2:** Obstetrics characteristics of study participants at Harar hospitals, Eastern Ethiopia, 2017 (*N* = 425)

Variables		Frequency	Percentage (%)
Parity	Primipara	136	32.0
	Multipara	289	68.0
Antenatal care follow-up	Yes	303	71.3
	No	122	28.7
Place of ANC follow-up (*n* = 303)	Health center	221	73.6
	Hospital	82	26.4
Number of ANC visit (*n* = 303)	< 4	233	76.9
	≥ 4	70	23.1
Reason to deliver in the hospital	Planned	265	62.4
	Referred	160	37.6
Status of current pregnancy	Planned	379	89.2
	Unplanned	46	10.8
Companion during labor	Yes	89	20.9
	No	336	79.1
Episiotomy	Yes	134	31.5
	No	291	68.5
Length of labor (In hours)	< 12	266	62.6
	≥ 12	159	37.4
Sex of labor attendant	Male	213	50.1
	Female	212	49.9
Mode of delivery	SVD	224	52.7
	Instrumental assisted	106	24.9
	Cesarean section	95	22.4
Maternal outcome	Normal	370	87.1
	With complication	55	12.9
Length of hospital stay	≤ 24 h	305	71.8
	> 24 h	120	28.2

### Status of respectful maternity care

**Fig. 2:**
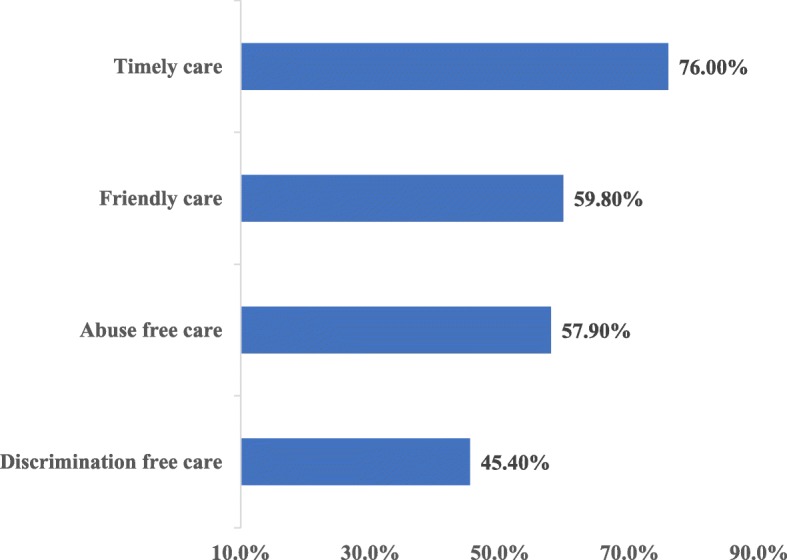
Status of respectful maternity care by categories at Harar hospitals, Eastern Ethiopia, 2017 (*N* = 425)

Overall, only 38.4% (95%; CI: 33.7, 42.0%) of the women received RMC. Among the participants, ~ 45% received discriminative free care (Fig. 2). Of the participants, 17.6% didn’t receive information about pain relief measures and 16.5% complained that HCPs did not show any concern and empathy. One-third (33.2%) of women reported that the HCPs did not respond to their needs. A significant number (14.8%) of women complained that some HCPs shouted at them during childbirth. Nearly, one-fifth (18.6%) were insulted due to the presence of a birth companion and personal attributes (Table 3).

**Table 3:** Categories and types of respectful maternity care reported by women during childbirth in Harar hospitals, Eastern Ethiopia, 2017 (*N* = 425)

Categories of respectful and abusive free care	Items of respectful and abusive free care	Yes, N (%)
Friendly care	I felt that healthcare workers cared for me with a kind approach.	368 (86.8)
	Healthcare workers treated me in a friendly manner.	369 (86.8)
	The healthcare providers were talking positively about pain and relief.	350 (82.4)
	The health worker showed his/her concern and empathy.	355 (83.5)
	All healthcare workers treated me with respect as an individual.	354 (83.3)
	The healthcare workers speak to me in a language that I can understand.	374 (93.9)
	The healthcare providers called me by my name.	357 (84.0)
Abusive free care	The healthcare workers responded to my needs whether or not I asked.	284 (66.8)
	Some healthcare providers slapped me during delivery for different reasons.	48 (11.3)
	Some health workers shouted at me because I haven’t done what I was told to do.	63 (14.8)
Timely care	I was kept waiting for a long time before receiving services.	53 (12.5)
	Service provision was delayed due to the health facilities’ internal problem).	69 (16.2)
Discrimination-free care	Some of the health workers do not treat me well because of some personal attribute.	57 (13.4)
	Some health workers insulted me and my companions due to my personal attributes.	79 (18.6)
	I was allowed to practice cultural rituals in the facility.	235 (55.3)

### Factors associated with respectful maternity care

After controlling confounding using multivariable analysis five variables: delivering at a private hospital, having ANC follow-up, planned pregnancy, labor attended by male providers, and normal maternal condition after delivery were significantly associated with RMC. The odds of RMC were 2 times [AOR: 2.3, 95% CI: 1.25, 4.07] higher among women who delivered at private hospitals as compared to women who delivered in public hospitals. The odds of RMC were almost 2 times [AOR: 1.8, 95% CI: 1.10, 3.20] higher among women who had ANC follow-up. Likewise, planned pregnancy increases the odds of RMC by three-fold [AOR: 2.9, 95% CI: 1.19, 6.92]. The odds of RMC were two times [AOR: 1.8, 95% CI: 1.14, 2.77] higher among women attended by male providers as compared to women whose labor attended by female providers. Moreover, the odds of RMC were 2 times [AOR: 2.3, 95% CI: 1.13, 4.83] higher among women whose childbirth process ends up without complications (Table 4).

**Table 4:** Bivariate and multivariable logistic regression result for factors associated with respectful maternity care in Harar hospitals, Eastern Ethiopia, 2017 (*N* = 425)

Variables	Respectful Maternity Care		COR (95% CI)	AOR (95% CI)
	Yes N (%)	No N (%)		
Place of delivery				
Public hospital	127 (35.0)	236 (65.0)	1	1
Private hospital	36 (58.1)	26 (41.9)	2.6 (1.49, 4.45)	2.3 (1.25, 4.07)^a^
Residence				
Urban	97 (44.5)	121 (55.5)	1.7 (1.15, 2.55)	1.1 (0.63, 2.0)
Rural	66 (31.9)	141 (68.1)	1	1
Educational status				
No formal education	73 (33.5)	145 (66.5)	0.7 (0.46, 1.18)	1.2 (0.62, 2.12)
Primary	46 (46.5)	53 (53.5)	1.3 (0.73, 2.19)	1.6 (0.87, 2.93)
Secondary and above	44 (40.7)	64 (59.3)	1.00	1.00
Parity				
Primipara	60 (44.1)	76 (55.9)	1.4 (0.94, 2.16)	1.3 (0.80, 2.02)
Multipara	103 (35.6)	186 (64.4)	1	1
Antenatal care visit				
Yes	133 (43.9)	170 (56.1)	2.4 (1.50, 3.84)	1.8 (1.10, 3.2)^a^
No	30 (24.6)	92 (75.4)	1.00	1.00
Status of pregnancy				
Planned	156 (41.2)	223 (58.8)	4.0 (1.70, 8.94)	2.9 (1.19, 6.92)^a^
Unplanned	7 (17.5)	39 (84.8)	1	1
Reason to deliver here				
Planned	112 (44.4)	140 (55.6)	1.6 (1.04, 2.36)	1.5 (0.86, 2.43)
Referred	51 (29.5)	122 (70.5)	1.00	1.00
Sex of the health care provider				
Male	91 (42.7)	122 (57.3)	1.5 (0.98, 2.15)	1.8 (1.14, 2.77)^a^
Female	72 (34.0)	140 (66.0)	1	1
Maternal condition after delivery				
Normal	151 (40.8)	219 (59.2)	2.5 (1.26, 4.84)	2.3 (1.13, 4.83)^a^
With complication	12 (21.8)	43 (78.2)	1.00	1.00
Presence of birth companion				
Yes	38 (42.7)	51 (57.3)	1.3 (0.78, 2.02)	1.3 (0.80, 2.22)
No	125 (37.2)	211 (62.8)	1.00	1.00

^a^ Statistically significant at *P* < 0.05

## Discussion

This study revealed that 45, 58, 60 and 76% of women received discriminative free, abusive free, friendly and timely care respectively. Overall, 38% of women received RMC. Delivering at a private hospital, having ANC follow-up, planned pregnancy, labor attended by male providers, and normal maternal condition after delivery were significantly associated with RMC.

In this study, merely 38.4% of women received RMC. This finding is higher than the study conducted in Addis Ababa (21%) [7] and lower than the studies conducted in public hospitals of Ethiopia (66%), Bahir Dar, Ethiopia (57%), Uttar Pradesh, India (43%), Tanzania (85%), and Kenya (80%) [22, 24, 25, 33, 34]. This incongruity may be due to study period variation, participant’s level of understanding about the service, educational and socio-economic status of the participants, service quality, and the ability of participants to report D&A. Likewise, it may be due to the normalization of D&A, client flow, sociocultural difference, and adherence of hospitals with women-friendly amenities.

Delivering at private hospitals is associated with RMC. Women who were delivered at private hospitals were two times higher to report, the service as reverential and cordial. This is likely due to the fact that in private hospitals, services are relatively luxurious and reachable to those of a higher wealth index. As a result, there is less chance of congestion with more concentrating service delivery due to the lower healthcare provider to patient ratio.

Attending the recommended ANC follow-up is linked with women’s pleasure in a study conducted in southern Ethiopia [5]. Similarly, in this study women who had ANC follow-up increase the odds of RMC by two-fold. The probable reason may be due to the client’s adaptation with the services and close relations with the HCPs during the ANC follow-up, which is crucial in building confidence in the service provided in the facility.

Evidence showed that the desired pregnancy upsurges women’s level of contentment and recognize the service provided in hospitals as reverential [35]. Correspondingly, in this study planned pregnancy increases the odds of RMC by three-fold. The possible reason may be, as pregnancy is desired and intentional; the mother receives continuous emotional support from her husband and families, which improves the outcome of childbirth. In addition, utilization of maternal health services also increased that helps her to be familiarized with the service providers, reduce depression and increase the mother’s attitude to perceive the care as courteous.

Labor attended by male HCPs is associated with RMC. The odds of RMC were two times higher among women whose labor attended by male providers. This finding is in line with the study conducted at public hospitals of Ethiopia in which, male HCPs were more likely to engage in the implementation of RMC [25]. This is challenging to explain but it might be due to the natural inclination to opposite-sex rather than the quality of care provided.

Normal maternal condition after childbirth was associated with RMC. The odds of RMC were two-fold higher among women whose childbirth process ends up without complications as compared to their counterparts. This is aligned with the studies conducted in Ethiopia [24, 36]. This could be due to the fact that those women who faced difficulties during labor are highly risky to develop post-partum blues and depression. In addition, those mothers who develop complications are admitted and stayed for an extended period of time without the full support of the families. Besides, they may feel that they develop such complications due to the poor quality of the service and perceive the service as offensive.

Despite the study follows scientific methodological approaches rigorously, it may have the following limitations: since the data were collected within the hospital environment there may be social desirability bias and fear of reporting abusive care. To minimize this bias, the data were collected in a private room within the hospital setup. The other limitation is that meanwhile the data were collected in the early postpartum period some women were fatigue to respond some questions.

## Conclusion

In general, only four out of ten women received RMC. Delivering at private hospitals, having ANC follow-up, planned pregnancy, labor attended by male providers, and normal maternal conditions were significantly associated with RMC. Providing a women-friendly, abusive free, timely and discriminative free care are the pillars to improve the low uptake of institutional delivery in Ethiopia. Hence, the involvement of multidisciplinary stakeholders is needed to deliver respectful and compassionate care for childbearing mothers. Furthermore, great emphasis should be given to make the service client-centered. Further large-scale community-based studies supplemented with qualitative data are needed to identify the barriers of RMC.

## Data Availability

The datasets used and/or analysed during the current study are available from the corresponding author on reasonable request.

## Abbreviations

ANC: Antenatal Care
D&A: Disrespect and Abuse
HCPs: Health Care Providers
MMR: Maternal Mortality Ratio
RMC: Respectful Maternity Care
